# Einkorn genomics reveals ancient roots of domesticated wheat

**DOI:** 10.1016/j.xgen.2023.100406

**Published:** 2023-09-13

**Authors:** Muhammad Arslan Mahmood, Shahid Mansoor

**Affiliations:** 1 Plant Sciences Division, Research School of Biology, The Australian National University, Canberra, ACT 2601, Australia; 2 International Center for Chemical and Biological Sciences (ICCBS), University of Karachi, Karachi, Pakistan

## Abstract

Wheat is an important staple food crop that underwent complex genome duplications. During domestication, genetic changes occurred, improving modern wheat, but understanding its phylogenetic history has been lacking. Mahmood and Mansoor discuss a recent publication demonstrating the evolutionary history of domesticated wheat (*Triticum* monococcum), providing opportunities for advancements in cereal improvement.

## Main text

**Figure 1:**
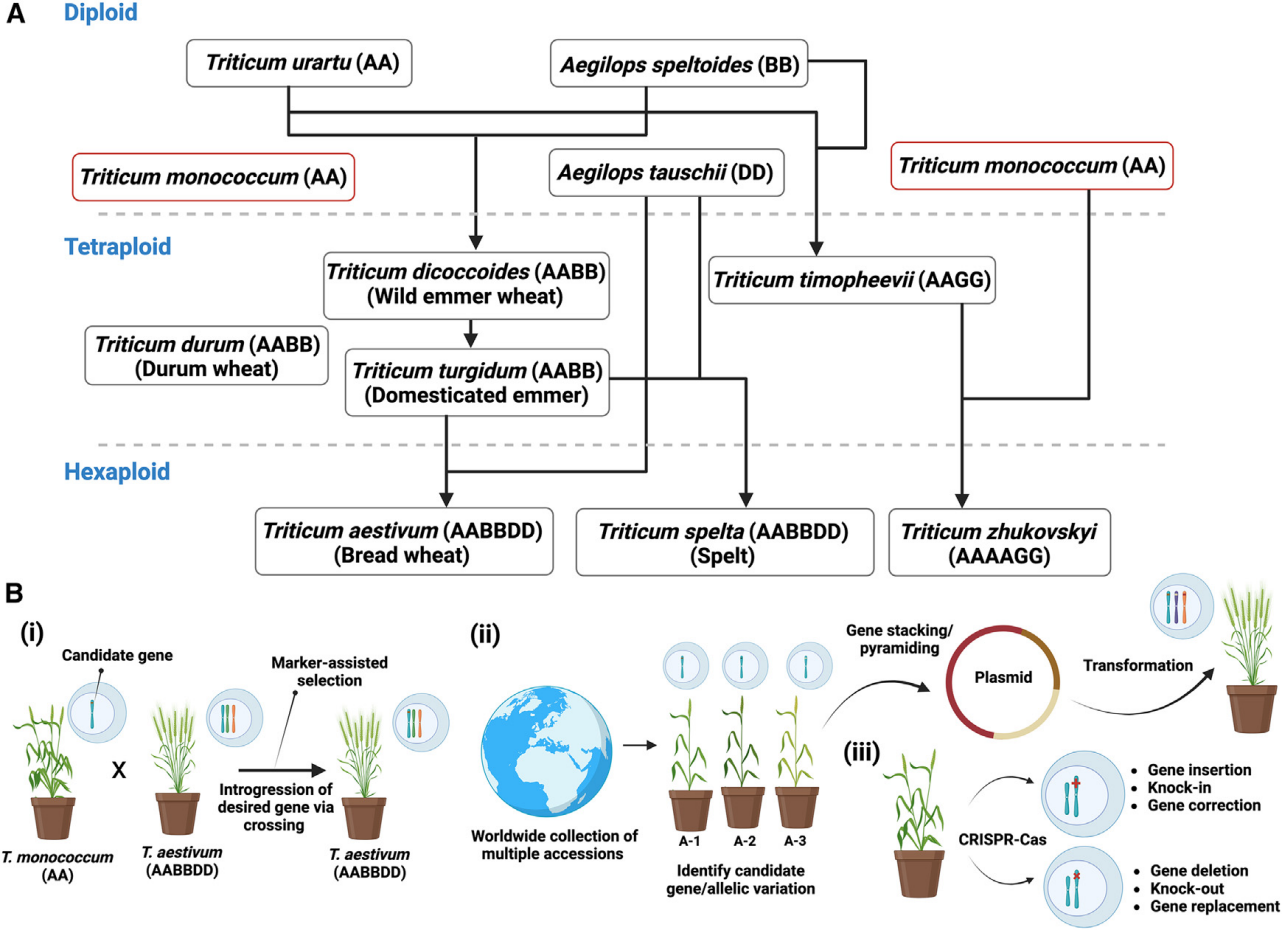
The ancestry of modern bread wheat and strategies for achieving significant agronomic traits from einkorn wheat genomic resources (A) Evolutionary relationship of wheat and its ancestors. (B) Options for crop improvements. (i) Candidate genes, such as resistance genes, can be introgressed into hexaploid wheat through breeding, which can further be expedited using marker-assisted selection. (ii) Acquisition of diverse wheat progenitors can be collected, and candidate genes/allelic variation identified by employing high-throughput sequencing techniques. Promising candidate genes can be utilized for the hexaploid, as well as tetraploid, wheat improvement. (iii) Alternatively, this panel diversity can potentially utilize a CRISPR-Cas targeted editing system for genome editing.

Domestic wheat (*Triticum aestivum*) is one of the most important crops consumed worldwide. To ensure global food security by 2050, wheat production must be increased sustainably by over 50%.^1^ Modern allo-hexaploid wheat originated from two polyploidization events. The first event involved tetraploidization, a doubling of a diploid genome, in which emmer wheat, *T. turgidum* (AABB), formed by the hybridization of the wild species *T. urartu* (AA) and a presumed extinct species *Aegilops speltoides* (BB) around 0.5 million years ago. A second event in which tetraploid wheat (AABB) hybridized with the wild diploid species *A. tauschii* (DD) resulted in the hexaploid *T. aestivum* genome (AABBDD)^2^ (Figure 1).

Despite increased adaptability to diverse environments, attributed to polyploidization, hexaploid wheat suffered a reduction in genetic diversity during domestication. However, its wild progenitors have maintained genetic diversity, utilized by breeders and researchers to develop high-yielding and elite wheat cultivars.^3^ Furthermore, high-throughput sequencing of wild relatives of wheat have expedited the productivity of trait improvement in modern hexaploid wheat.^4^ Population genomics and pan-genome analyses demonstrate that hybridization had an important role in enhancing genetic diversity in wheat.^5^ However, to maximize the genetic potential of wheat, knowledge and understanding of the structure and function of its genome, as well as relationship with its wild progenitor species, are required.

Ahmed et al.^6^ described high-quality 5.2-Gb genome assemblies including complete assembled and gap-free centromeres of einkorn (*T. monococcum*) wheat by employing a combination of PacBio circular consensus sequencing, chromosome conformation capture, and optical mapping. Einkorn wheat has a diploid genome (AA) and is resistant to pests and diseases and can also resist other stresses such as drought and low-fertility soil.

They assembled the centromeres of both wild and domesticated einkorn wheat without any sequence gaps and discovered that einkorn centromeres are notably flexible, devoid of tandem repeats. Analyses of wild and domesticated einkorn wheat accessions showed similar chromosomal segments adjacent to the functional centromeres, but the sequence collinearity is low or absent, providing evidence of ancient and recent centromere shifts, driven by transposon activity and structural rearrangements. Furthermore, the sequencing of 219 einkorn accessions reveals that einkorn wheat has gone through complex hybridization and introgression after its initial domestication and subsequent spread throughout Central Asia and Europe.

The wild and domesticated einkorn wheat genome shares a high degree of collinearity with the A-genome donor (*T. urartu*) of polyploid pasta (*T. durum*) and hexaploid bread wheat, providing an opportunity to deploy einkorn wheat as a model species for trait discovery that could lead to hexaploid wheat improvement. They further annotated approximately 32,000 genes on the 7 pseudomolecules of wild and domesticated einkorn wheat that closely resemble with the corresponding genes on subgenome A chromosomes in bread wheat.

Moreover, Ahmed et al. mapped and cloned the *tiller inhibition* (*tin3*) gene, which is a significant shoot architecture trait of many cereal crops. This gene was identified as a result of an EMS (ethyl-methanesulfonate)-induced point mutation screen in einkorn wheat, where mutants exhibited reduced tiller number. They also identified the *tin3* gene ortholog in barley (*Cul4*) and hexaploid wheat. However, in hexaploid wheat, *tin3* mutants contained point mutations in one or two homeologs but exhibited normal tillering, while triple homeologous mutants had reduced tiller number. This indicates that around 1% of the modern wheat (*T. aestivum*) A-genome directly originated from einkorn wheat. This finding highlights that einkorn wheat can be utilized as a genomic resource for tetraploid and hexaploid wheat improvement.

The findings of this study provide a genomic resource for the study of and improvement of wheat and related cereals. Identifying and transferring new candidate genes, such as those responsible for pest, disease resistance, and drought tolerance traits, from einkorn into polyploid wheat holds great promise for crop improvement. Natural and artificial introgressions of einkorn into hexaploid wheat have yielded successful results in conferring resistance against fungal diseases. For example, the *Sr35* gene, cloned from *T. monococcum*, provides resistance to the stem rust strain Ug99,^7^ a major wheat pathogen. This gene is absent in the A-genome of diploid donor and in hexaploid wheat but is effective when transferred from *T. monococcum*. In addition, *Yr34* has been transferred to polyploid wheat from *T. monococcum* and conferred resistance against stripe rust.^8^

Moreover, sequencing the einkorn genome provides a complete picture of functional and structural organization of Triticeae centromeres that can be exploited for breeding programs of modern wheat. To utilize complete genetic diversity of einkorn in modern wheat, the most common route involves hybridization between cultivated tetraploid wheat and *T. monococcum* followed by chromosome doubling to develop synthetic hexaploid wheat.

Recently, D-genome species, *A. tauschii* accessions were sequenced and compared with the hexaploid wheat D-genome, identifying a rare lineage of *A. tauschii* that contained candidate genes associated with resistance to pests and diseases.^9^ The synthetic hexaploid approach in combination with high-throughput sequencing data of wheat wild progenitors promises an effective and fast-track novel germplasm development approach for breeding.

Direct hybridization between hexaploid wheat and einkorn is also possible. This approach usually requires embryo rescue but has the advantage that it does not interrupt desirable allele combinations in the polyploid wheat. Notwithstanding, the product of these wide crosses need backcrossing to domesticated cultivars to eliminate undesired agronomic traits and restore optimal end-use qualities. However, if haplotypes underlying essential traits identified in einkorn wheat, this would mitigate a limitation in breeding wheat. Such haplotypes can be marked with molecular markers for accelerated delivery into modern wheat by combining marker-assisted selection with rapid generation advancement (Figure 1). This provides an opportunity for breeders to use strategic introgression of the new resistance haplotypes to develop specific-pathogen-resistant wheat varieties. Einkorn wheat can also be used to identify and clone recessive genes, of which the phenotypic effects masked in a polyploid. Improving such gene-level knowledge could allow for gene stacking/pyramiding through transgenesis or by generating desired allelic variations via CRISPR-Cas genome editing^10^ (Figure 1). In summary, einkorn wheat offers an opportunity to develop functional genomic resources for cereals with the knowledge gained from this diploid species ready to be transferred to tetraploid pasta wheat and hexaploid bread wheat.
